# Structural constraints revealed in consistent nucleosome positions in the genome of *S. cerevisiae*

**DOI:** 10.1186/1756-8935-3-20

**Published:** 2010-11-12

**Authors:** Christoforos Nikolaou, Sonja Althammer, Miguel Beato, Roderic Guigó

**Affiliations:** 1 Bioinformatics and Genomics Group, Centre for Genomic Regulation (CRG), Biomedical Research Park of Barcelona (PRBB), Barcelona, 08003, Catalunya, Spain; 2 Gene Regulation and Chromatin Group, Centre for Genomic Regulation (CRG), Biomedical Research Park of Barcelona (PRBB), Barcelona, 08003, Catalunya, Spain; 3 Department of Biology, University of Crete, 71409 Heraklion, Greece

## Abstract

**Background:**

Recent advances in the field of high-throughput genomics have rendered possible the performance of genome-scale studies to define the nucleosomal landscapes of eukaryote genomes. Such analyses are aimed towards providing a better understanding of the process of nucleosome positioning, for which several models have been suggested. Nevertheless, questions regarding the sequence constraints of nucleosomal DNA and how they may have been shaped through evolution remain open. In this paper, we analyze in detail different experimental nucleosome datasets with the aim of providing a hypothesis for the emergence of nucleosome-forming sequences.

**Results:**

We compared the complete sets of nucleosome positions for the budding yeast (*Saccharomyces cerevisiae*) as defined in the output of two independent experiments with the use of two different experimental techniques. We found that < 10% of the experimentally defined nucleosome positions were consistently positioned in both datasets. This subset of well-positioned nucleosomes, when compared with the bulk, was shown to have particular properties at both sequence and structural levels. Consistently positioned nucleosomes were also shown to occur preferentially in pairs of dinucleosomes, and to be surprisingly less conserved compared with their adjacent nucleosome-free linkers.

**Conclusion:**

Our findings may be combined into a hypothesis for the emergence of a weak nucleosome-positioning code. According to this hypothesis, consistent nucleosomes may be partly guided by nearby nucleosome-free regions through statistical positioning. Once established, a set of well-positioned consistent nucleosomes may impose secondary constraints that further shape the structure of the underlying DNA. We were able to capture these constraints through the application of a recently introduced structural property that is related to the symmetry of DNA curvature. Furthermore, we found that both consistently positioned nucleosomes and their adjacent nucleosome-free regions show an increased tendency towards conservation of this structural feature.

## Background

Recent studies have suggested that the nucleosomal organization of eukaryotic genome, should not be considered a mere obstacle but rather a vital component of fundamental molecular processes, such as transcriptional regulation [1,2] or replication [3,4]. However, the principles that specify the location of nucleosomes along the DNA sequence remain largely elusive. In many cases, the specific positioning of nucleosomes or even their selective disruption [5] has been shown to be driven by underlying sequence elements, but the extent to which DNA guides the positioning remains an open question.

Genomewide nucleosomal maps may assist in the better understanding of the process. Yuan *et al*. [6] first defined the nucleosomal positions for a significant portion of the yeast genome using micrococcal nuclease digestion followed by microarray hybridization. Lee *et al*. [7] used the same technique at a higher resolution to map nucleosomes along the entire yeast genome, and Shivaswamy *et al*. [8] defined the yeast nucleosome positions under two different conditions using high-throughput sequencing. Comparing the results of these studies has proved to be more complicated than expected. In a recent work, Stein and colleagues [9] examined independent nucleosomal datasets for the genome of *Saccharomyces cerevisiae *in an attempt to define the sequence prerequisites for nucleosome formation. They reported a limited degree of consistency between different datasets, and reached the conclusion that DNA sequence preferences have only small effects in the placement of nucleosomes *in vivo*. An independent study by Feng *et al*. [10] focused on two individual genomewide datasets [7,8]. They suggested that two distinct classes of nucleosomes of different stability coexist in the cell nucleus, but they failed to detect any sequence-specific preferences in either of the two classes.

Defining the sequence properties of nucleosomal DNA has been the focus of a number of studies, with the main scope being the prediction of nucleosome positioning [11-16]. Regardless of the computational approaches undertaken, which may vary from likelihood models [13,16] to comparative genomics [12] and supervised learning strategies [14,15], the combined results reach two basic conclusions. Firstly, that only a subset of the cell nucleosome positions may be predictable and secondly, that the sequence constraints of nucleosomal DNA, if any, appear to be very weak. Even recent studies that have attempted to establish a clearer connection between the underlying sequence and the positioning of nucleosomes in the light of new experimental evidence [11,17-20] have not attempted to provide a concise framework for what drives nucleosome positioning on DNA, other than the well-reported, ubiquitous dinucleotide periodicities. Overall, the observations made by various research groups using both experimental and theoretical approaches imply that there are few regions in the genome in which the nucleosomal landscape is consistent across the cellular population, and that the majority of the nucleosomes are stochastically positioned. Primary sequence conservation seems to be of little assistance in attempts to define the population of consistently positioned nucleosomes.

The idea for the existence of a subset of 'important' nucleosomes that statistically guide the overall positioning of the bulk set is not new. It was introduced in early works by Kornberg and Stryer [21,22] and later studies [23,24] have further elaborated this concept. In a recent work, Reynolds *et al*. [25] proposed a very efficient nucleosome-positioning model that supports statistical positioning. They provided a concise theoretical framework for the discrimination of nucleosome-bound versus nucleosome-free regions (NFRs), and iterated the observation of only a small fraction of the complete nucleosomes being well positioned. However, they did not examine whether the used patterns are under any sort of constraint.

In this study, we focused on precisely this aspect, aiming to identify the specific constraints that are expected to be underlying well-positioned nucleosomes. We first defined a subset of consistently positioned nucleosomes in the yeast genome as primary candidates for playing an organizing role in chromatin structure. We show that, in contrast to the surprising absence of sequence conservation, these nucleosomes are under strong structural constraints that are related to DNA curvature. Furthermore, we show that these structural constraints are retained in consistent nucleosomes in contrast to the bulk nucleosomes, in which structural tendencies are weaker and exhibit greater flexibility. We therefore propose that statistical positioning may partially impose secondary structural constraints within consistent nucleosome positions.

## Results and discussion

### Consistent nucleosomes in the genome of *S. cerevisiae*

A number of studies have attempted to define the complete set of nucleosome positions along the yeast genome [7,8,13,23,24]. The final output of these studies varies. Lee *et al*. [7] applied a hidden Markov model on their raw data, which resulted in a set of genomic coordinates for well-positioned nucleosomes. Shivaswamy *et al*. [8] directly provided sequence coordinates for the inferred nucleosome positions. Mavrich *et al*. [23] provided the densest of all datasets, with more than 65,000 nucleosome positions (in the form of genomic coordinates of the pseudodyad axes), but these belonged to a mixed population originating from different experimental approaches (sequencing and tiling arrays), which in addition included hypothetical nucleosomes (that is, positioned by the authors based on general occupancy assumptions). Kaplan *et al*. [13] assigned a score to each nucleotide in the yeast genome, reflecting the *in vitro *affinity of the underlying sequence for histone binding. By contrast, Zhang *et al*. [24] provided only the raw datasets, making it difficult to include their inferred positions in a comparative study.

We thus chose to directly compare the datasets of Lee *et al*. [7] and Shivaswamy *et al*. [8], because they both consist of homogeneous datasets that span the complete genome of *S. cerevisiae*, and to use the data of Mavrich *et al*. [23] and Kaplan *et al*. [13] for further validation of our results. The sparseness of unaddressed sequence space in the datasets of Lee *et al*. [7] and Shivaswamy *et al*. [8] makes the comparison between them less complicated (see below). In addition, both datasets consist of positions with reference to genomic coordinates, providing a consistent platform for direct comparison. The analysis was carried out as follows.

We connected each of the experimental calls of Lee *et al*. [7] (40,089 nucleosomes) with its closest corresponding well-positioned nucleosome in the dataset of Shivaswamy *et al*. [8] (49,043 nucleosomes). For every such pair, we calculated the percentage of overlap, expressed as the percentage of the intersection over the union of their corresponding lengths. We then analysed the distribution of these overlap values (see Additional File 1). We found that 31,234 nucleosomes (78%) of the Lee *et al*. [7] dataset had at least one nucleotide overlap with one of 33,146 nucleosomes (68%) of the Shivaswamy *et al*. dataset [8]. In the analysis, we excluded non-overlapping segments to avoid biases originating from local discrepancies between the two datasets (for example, sequencing preferences, experimental biases, or gaps). We thus confined our study to the overlapping nucleosomes between the two datasets. Of these overlapping positions, only 9.8% of the cases (3,061 nucleosomes) had an overlap exceeding 0.95, and 17.8% of the cases (5,560 nucleosomes) had an overlap greater than 0.90. These overlaps are still much larger than expected by chance. To test the significance of these values, we performed 1,000 simulations of two random experiments, with an equal number of nucleosomal calls (see Additional File 1). The overlap values between two random experiments followed a Poisson distribution, which was significantly different from the observed distribution (*P *< 10^-3^, Kolmogorov-Smirnov test). In fact, an overlap ratio of 0.95 was not encountered in any of the 1,000 simulations. We should note here that the positions by Shivaswamy *et al*. [8] were provided at single-nucleotide resolution, whereas those of Lee *et al*. [7] were given with a resolution equaling that of the chip used, which was four nucleotides. The latter corresponds to ~2.5% of the nucleosome length, which, taken on either side, roughly translates to a 5% uncertainty value for each nucleosome position. We therefore believe that a value of 95% represents the maximum possible overlap, given the combined resolution of the datasets and thus opted for the overlap ratio of 0.95 to define well-positioned nucleosomes that were consistent between the two sets. As these are expected to have a stable position across all cells in the population, we shall refer to them as 'consistent nucleosomes' hereafter.

### Consistent nucleosomes are well positioned *in vivo *and *in vitro*

A direct comparison of the two previously discussed datasets and their overlapping subsets was then crossvalidated using the scores of Shivaswamy *et al*. [8], Mavrich *et al*. [23] and Kaplan *et al*. [13]. Initial validation of the consistent nucleosomes was conducted through direct comparison with *in vivo *and *in vitro *hybridization scores. We used the initial *in vivo *scores provided by Shivaswamy *et al*. [8] for each of their nucleosome calls. We then compared the scores of consistent nucleosomes with non-overlapping nucleosomes; that is, those that had zero overlap between the two sets. Scores for nucleosomes with no overlap were significantly lower than for those with high overlap values (see Additional File 1). Because these scores were directly linked to the initial raw experimental signal, the observed differences may be an indication that consistent nucleosomes are positioned more strongly *in vivo*.

Mavrich *et al*. [23] also provide a score for each inferred position. This is an occupancy measure based on the mode-normalized occupancy across all of their four sequencing datasets, ranging from 0 to 100; a mean score of 100 represents nucleosomes found at the same position in all four replicate datasets. To further validate our dataset of consistent nucleosomes, we analyzed the cases that had > 0.95 overlap with the dataset of Mavrich *et al *(~66,000 nucleosomes), and plotted the distributions of mean occupancy for the complete datasets as their overlapping subsets (see Additional File 1). We found that mean occupancy increased sharply as we moved from the initial complete dataset of Mavrich *et al*. [23] to two-set overlaps, reaching a mean of 93 in the case of the three-set overlap (segments from Lee *et al*. [7], Shivaswamy *et al*. [8] and Mavrich *et al*. [23] that were overlapping in > 0.95 of their combined lengths). In fact, none of the 3061 consistent nucleosomes, defined as the overlaps between Lee *et al*. [7] and Shivaswamy *et al*. [8], showed an overlap of < 0.96 with a corresponding segment of the denser Mavrich *et al*. [23] dataset, an additional indication that they comprise a subset of highly reproducible positions.

In a recent work, Kaplan *et al*. [13] attempted to decouple the intrinsic sequence preferences of nucleosomes from the combined action of all influencing factors, and thus provided a measure for the *in vitro *affinity of the underlying DNA for nucleosome formation. Although this is expected to correlate well with *in vivo *positioning, we examined possible differences between consistent and non-overlapping nucleosomes under *in vitro *conditions using the model scores as calculated by Kaplan *et al*. [13] (see Methods for details). We found a significant enrichment of model scores for consistent nucleosomes compared with zero overlap nucleosomes, as expected (see Additional File 1).

Taken together, these results verify our hypothesis that well-defined nucleosomes are positioned more strongly both *in vivo *and *in vitro*.

### Sequence conservation in consistent nucleosomes

**Figure 1:**
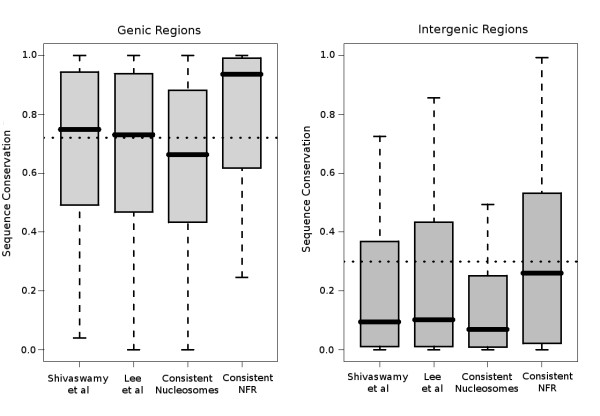
**Distribution of average sequence conservation (expressed as seven-way phastCons) for the complete nucleosome datasets, 3,061 consistent nucleosomes and 1,099 inter-nucleosomal consistent nucleosome-free regions (NFRs)**. Average conservation was calculated as the sum of PhastCons values for a given region divided by its total length. **(A) **Genic regions partition (n = 5,770). Segments overlapping 5,770 yeast genes from Shivaswamy *et al*. [8] (42041 nucleosomes), Lee *et al*. [7] (31,739 nucleosomes), consistent nucleosomes (2,449 cases), consistent NFRs (879 cases). **(B) **Intergenic regions partition (5,442). Segments included in 5,442 yeast intergenic regions from Shivaswamy *et al*. [8] (7,002 nucleosomes), Lee *et al*. [7] (8,350 nucleosomes), consistent nucleosomes (612 cases), consistent NFRs (220 cases). Dotted horizontal lines correspond to the mean conservation of genic and intergenic regions respectively, calculated as the average over the sum of their lengths.

The focus of this work was the investigation of sequence constraints characterizing consistent nucleosomes. We used phastCons sequence conservation scores [26] (see Methods), as a measure of evolutionary conservation between seven species of the genus *Saccharomyces*, to calculate the average sequence conservation for the genomic regions under study. To avoid biases originating from the background genomic composition, we partitioned all datasets into genic and intergenic nucleosomes. Those falling within genes were treated separately from those occupying non-coding genomic space. Conservation of bulk nucleosomes in the two complete genome datasets [7,8] is comparable with background conservation in the yeast genome (0.71 for genic nucleosomes, compared with 0.73 for yeast genic regions). Interestingly, consistent nucleosomes show overall lower conservation than the genomic background for both genic and intergenic partitions of the genome (Figure 1). This finding is somehow counterintuitive. Consistent nucleosomes were shown to have enriched affinity for histone molecules as suggested by both *in vivo *and *in vitro *experiments and to share particular positional preferences (see Additional File 1). These facts combined would imply an increased functional role for the underlying sequences, which does not easily comport with the observed lack of sequence conservation.

A number of studies [6,23,27] have pointed out the role of NFRs in gene regulation. Such regions are usually enriched in regulatory *cis*-acting binding elements for transcription factors that exclude the positioning of nucleosomes and could thus indirectly affect the organization of chromatin. These NFRs are therefore expected to be under specific sequence constraints, and related to the positioning of nucleosomes. To test whether such regions may be related to our consistent nucleosomes (as defined above), we extracted the genomic regions that fell between two adjacent consistent nucleosome segments We defined as 'adjacent' two consistent nucleosomes separated by a distance less than the size of a nucleosome and termed their intervening Nucleosome-Free Regions as 'consistent NFRs'. The set of these regions consisted of 1,099 genomic segments with an average length of ~40 nucleotides. The small percentage (~10%) that had a length of > 100 nucleotides suggested that they probably represent natural nucleosome linker sequences; that is, short spacers between two juxtaposed well-positioned nucleosomes. There has been evidence that NFRs of greater length may accommodate specific nucleosomes, whose unstable nature obstructs their direct observation [28]. We thus restricted our definition of consistent NFRs to consider only regions shorted than a full nucleosome. The relative enrichment (1,099 pairs out of 3,061) of such short linkers occurring between consistent nucleosomes reflects an additional property of the latter, namely, their preference to appear in pairs of dinucleosomes (see also Additional File 1).

As expected, consistent NFR segments showed increased sequence conservation. PhastCons scores calculated for these regions showed an average sequence conservation above the yeast genomic background for both genic and intergenic regions. Although strong sequence constraints are known to exist in these regions, this extremely high conservation of consistent NFR suggests an increased importance at various levels. Overall, the 1.099 defined segments represented less than 0.5% of the length of the total genome, and even if this is an underestimate due to restricted coverage of the analyzed datasets, their extent is unlikely to exceed 1% of the total genome size. On the one hand, the extremely high conservation of these sequences indicates multiple roles, which may include the guiding of nucleosome positioning (see below). On the other hand, their overall rarity suggests that signals of a different type (possibly lying within nucleosomes) are probably also necessary even though they may not be reflected in the primary sequence conservation.

### Possible role of consistent nucleosomes in gene regulation

**Figure 2:**
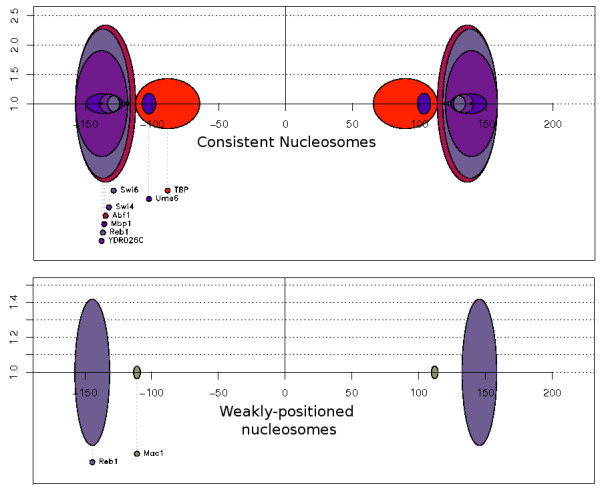
**PEAKS **[29]**output patterns**. **(A) **3,061 consistent nucleosomes with > 95% overlap between two independent experimental datasets [7,8]; **(B) **3,288 weakly positioned nucleosomes with < 5% overlap. Both sets were centrally aligned around the position of the assumed pseudodyad axis, representing 0 in the *x *axis. Both forward and reverse complements of each sequence were included in the analysis. Colored bubbles correspond to positional biases of significant regulatory motifs as defined by Harbison *et al*. [30]. Each significant motif is represented as an oval, colored according to an arbitrary internal legend. The width of each oval corresponds to the range of the position of significance, and the height is the relative motif signal (RMS). The RMS is defined as the maximum number of sequences that contain the motif in the region over the number of sequences that contain the motif at the *P*-value cut-off level; that is, the random expectance of the motif occurring in that region in 1000 random sets (for example, the elongated red oval for Abf1 in (A) was found to a significant bias in positions 120 to 150 nucleotides away from the center of the nucleosome, and in 2.5 times more sequences than those expected under a random model, thus having an RMS value of 2.5). Notice the difference in the scale between the two panels, suggesting increased fold biases for the consistent nucleosomes. Images produced by PEAKS are reproduced here with the permission of the program's authors.

The proximity of consistent nucleosomes to highly constrained NFRs that carry regulatory elements suggests that stable nucleosome positioning may be functionally related to the nearby binding of transcription factors. In fact, NFRs that host transcription factor binding elements have been proposed to act as nucleosome positioning markers [6,23]. If this holds, positional preferences defining a mutual exclusion between transcription factor binding sites and nucleosomes would be expected. To test this, all consistent nucleosomes were centrally aligned and extended in both directions for 100 nucleotides. The resulting sequences (347 nucleotide long) were then analyzed with PEAKS [29], an application to identify significant motif positional biases in pre-aligned DNA sequences. We performed a PEAKS search of yeast-specific regulatory motifs as defined by Harbison *et al*. [30]. The results of this analysis revealed a significant over-representation of positional biases for regulatory motifs, which are predominantly confined to the flanking regions of consistent nucleosomes, in clear contrast with the absence of significant motifs in the centre of the sequence corresponding to the nucleosome (Figure 2). The biased motifs belonged to a number of transcription factors and had variable sequence compositions, with both AT-rich (TBP) and GC-rich (Ume-6, Reb1, Swi4) sites found among them, suggesting that these biases are partly independent from sequence composition and probably reflect more general structural or/and functional tendencies.

Identical analysis was performed on a set of nucleosomes that was defined as the low overlap (< 5% but at least one overlapping nucleotide) fraction of the two datasets [7,8]. This set comprised 3,288 segments obtained from the Lee *et al*. [7] study, which we termed 'weakly positioned nucleosomes'. Inspection of the PEAKS output suggested much weaker positional biases for consistent nucleosomes. Only a few significant motif biases that lacked specific positional preference with respect to the nucleosome position were present.

### Structural constraints of consistent nucleosomes

A number of studies have suggested the existence of a 'nucleosome code' [11-13,16,31], but existing models appear to explain the binding of only a part of the complete set of nucleosomes. However, genome-scale studies on nucleosome formation in more complex eukaryotes [32,33] have reported limited sequence constraints in nucleosome sequences. It remains to be resolved whether this apparent lack of constraint is only an artifact of limited knowledge or due to inherent attributes of the dynamics of the process.

The results presented thus far suggest an overall absence of primary sequence constraints in the form of sequence conservation even in well-positioned nucleosomes. Nonetheless, even under an assumed statistical model, some degree of constraint would be expected in those positions that are thought as instrumental for the overall nucleosome-positioning process. In the case of nucleosomes, which are particles involved in chromatin structure and conformation, it is plausible to expect that constraints may be better reflected within the DNA structure rather than in the primary sequence itself. We therefore introduce a concept of structural information, which is directly related to the expected curvature of a DNA sequence.

The importance of curvature in the formation of nucleosomes has been demonstrated by several studies [34-37]. The DNA sequence spooling the histone octamer is curved, and it is plausible to assume that specific curvature patterns may be advantageous for a nucleosome-forming sequence. Another important aspect of nucleosomes is their inherent symmetry. The 147 bp nucleosomal sequence wraps around the octamer in a symmetric manner, and its central part is associated with the octamer's pseudodyad axis, a region that has been shown to bear structural features distinguishing it from its flanking regions [34,38-40]. Starting from the above observations, we hypothesized that nucleosomal DNA sequences are likely to consist of: (i) two symmetrically curved regions flanking the central part, as DNA is expected to be curved in a similar manner on either side of the pseudodyad axis; and (ii) a region of higher flexibility associated with the pseudodyad axis, at which the two superhelical loops have to meet and spool the histone octamer in a more distorted conformation. If these properties are to be reflected in the underlying nucleosomal DNA structure, then a measure that efficiently quantifies them should reveal structurally related differences between nucleosome-binding and nucleosome-free sequences.

**Figure 3:**
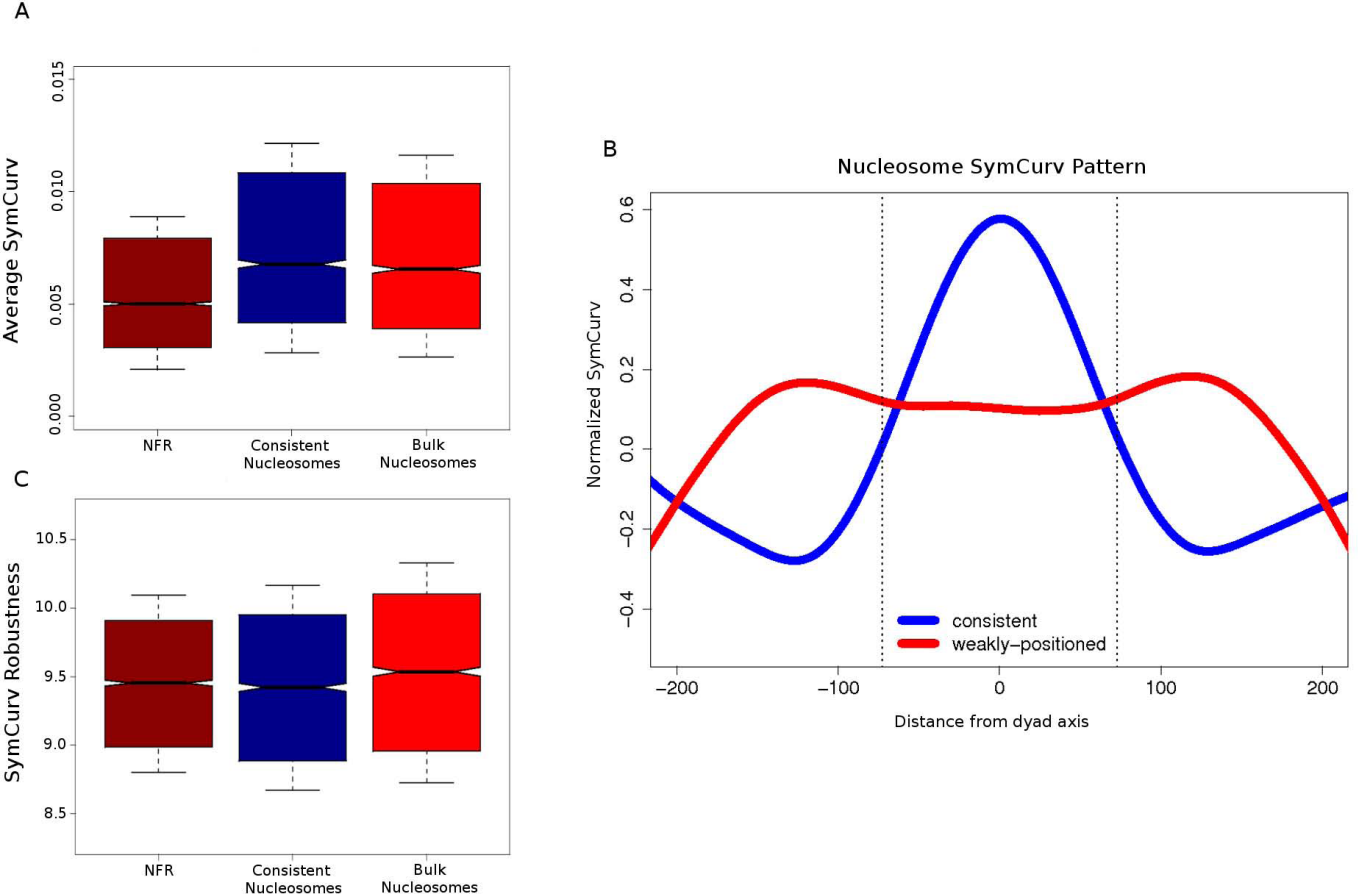
**Structural constraints in consistent nucleosomes reflected in SymCurv values. **(A) Distributions of SymCurv [41]values for 1,099 consistent nucleosome-free (NFR) sequences, 3,061 consistent nucleosomes and 3,288 weakly positioned nucleosomes in the form of box plots. One value was calculated for each sequence as the average SymCurv value over all nucleotides (see Methods). Solid boxes extend to cover the core 50% of the values around the median, whiskers extend to the further points, and notches extend to 1.58 of the interquartile range. Non-overlapping notches between two distributions suggest significant differences at 95% confidence interval. Values of consistent nucleosomes are significantly greater than both linker (t-test, *P *< 10-^16^,) and weakly positioned nucleosomal sequences (t-test *P *< 0.001). **(B) **Normalized SymCurv values for the central 447 nucleotides of consistent against weakly positioned nucleosomes. Both forward and reverse complements of each sequence were included in the analysis. Values were calculated here as the average SymCurv value over each position on a set of centrally aligned sequences (that is, the centre of the sequences was set to 0, and each nucleotide was assigned a coordinate relative to the centre), and were subsequently normalized against the overall average in the yeast genome (see Methods). Curves were smoothed with a running average on a window of 50 bp for better representation. Dotted vertical lines represent the boundaries of consistent nucleosomes. **(C) **Robustness of SymCurv values for consistent NFR, consistent nucleosomes and weakly positioned nucleosomes. Robustness was defined as the negative logarithm of average SymCurv variance in the set of one-nucleotide neighbors of each sequence. Low SymCurv robustness values for consistent nucleosomes versus weakly positioned nucleosomes (*t*-test *P *< 10^-7^) reflect increased constraints at the structural level. Similarly low values of consistent NFR sequences suggest that such constraints are also present in these sequences apart from those at the sequence level.

In a recent work [41], we introduced a structural property that quantifies the strength of the pattern described above. We implemented this concept ('symmetry of DNA curvature') in a suitably adjusted method (SymCurv), which permits us to attribute a symmetry of curvature score to each nucleotide on a given DNA sequence (see Methods for details). We calculated SymCurv for the complete genome sequence of *S. cerevisiae*, and went on to check whether consistent nucleosomes showed the expected tendency for high SymCurv values. The results are shown in Figure 3a. Consistent nucleosomes were found to have significantly higher SymCurv values compared with their adjacent linker sequences, whereas weakly positioned (unstable) nucleosomes (that is, those with < 5% overlap between the two sets) were shown to have significantly lower values than the consistent nucleosomes. A more thorough examination was conducted after centrally aligning all consistent and weakly positioned nucleosomes, and calculating the average SymCurv value over each nucleotide position with respect to the centre of the assumed nucleosome (Figure 3b). Consistent nucleosomes had the expected low-high-low SymCurv pattern, which reflects a strongly positioned nucleosome in the centre, flanked by two NFRs. Weakly positioned nucleosomes significantly deviated from this pattern, showing a wider peak with a 'fuzzier' shape with lower SymCurv values and with less pronounced boundaries. The SymCurv pattern for consistent nucleosomes is therefore suggestive of specific structural preferences related to nucleosome positioning, which are not reflected in the sequence conservation.

To better establish the existence of this structural constraint, we went on to test whether SymCurv values of consistent nucleosomes are susceptible to minor changes in the primary sequence. If structural constraints exist, it would be expected that small changes, even point mutations, would bring about significant changes in the structural profile of the sequence, thus the sequence will have low structural robustness. We formulated a simple measure of robustness for SymCurv, based on the variance of SymCurv values over all one-nucleotide neighbors of a given sequence (see Methods for details). A sequence under strong structural constraints is expected to exhibit high variance, and therefore its robustness will be low. We calculated SymCurv robustness values for the three sequence classes under study and plotted the corresponding distributions (Figure 3c). Consistent nucleosomes had robustness values similar to those NFRs, and were significantly lower than those of weakly positioned nucleosomes. The low robustness values of both consistent nucleosomes and NFRs are indicative of strong structural constraints, even though their profiles in terms of SymCurv values are significantly different. In the case of NFRs, these constraints coexist with those of the primary sequence as indicated by high sequence conservation, which may offer an explanation for their extremely high conservation, reflected in very high average phastCons values (Figure 2). By contrast, consistent nucleosomes also appear to be under structural constraints, but at the same time lack significant conservation at the level of primary sequence. This may be an additional indication for the existence of a weak structural code for nucleosome positioning, which is only indirectly connected to the DNA sequence.

## Conclusions

The existence of a DNA code for nucleosome positioning would impose a considerable informational load on any eukaryotic genome, guiding the positioning of only a subset of nucleosomes appears to be a more plausible hypothesis. Models for the statistical positioning of nucleosomes were proposed relatively early [21,22], but to what extent the whole process may be driven by a restricted subset of sequences was difficult to test before the accumulation of experimental data. Furthermore, there are multiple candidates for the role of the effector sequences for statistical positioning. NFRs with high sequence conservation and affinity for various transcription factors have been suggested to play an organizing role in the formation of nearby nucleosomes [6,7,16]. By contrast, well-positioned nucleosomes, occupying key positions close to the transcription start sites of genes, have also been reported to impose regular spacing of neighboring nucleosomes [23,42], a situation that resembles the 'parking lot' model proposed by Kiyama and Trifonov [43].

The question concerning the sequence properties of these nucleosome positioning elements is perhaps even more interesting than their distribution along the genome. Assuming a functional role even for a restricted part of the eukaryotic genome would imply the existence of some type of constraints that would reflect the importance of nucleosomal elements in fundamental cellular processes. Such constraints have remained elusive for the great majority of nucleosome sequences. Experimental evidence from higher eukaryotes [32,33] suggests an overall lack of sequence conservation for the majority of nucleosome sequences. On the other hand, a number of theoretical models for nucleosome-forming sequences [12,13,15,16,25] have indicated various sequence tendencies related to DNA composition and periodicities, but to what extent these properties are constrained (this being the prerequisite for any code) is a question that has not been addressed in full.

This work constitutes an attempt to provide evidence for the existence of a specific type of constraints in a subset of nucleosomes that share particular positional and structural preferences. Our observations, after a thorough comparison of published nucleosome datasets in the yeast genome, support a model of statistical positioning with only a restricted subset of the total nucleosomes being well positioned. Such consistent nucleosomes show a lack of sequence conservation compared with their adjacent NFRs, and there is a strong tendency for transcription factor binding sites to be found at their boundaries. Combined, these findings are strong indications for the NFRs being the organizing elements in the process of nucleosome positioning. According to this hypothesis, NFRs would provide the scaffold for the strong binding of other DNA-related proteins, allowing the remaining space to be bound by the histone octamers. However, a look, at the secondary structure of DNA shows that a concise nucleosome-positioning model is probably more complicated.

**Figure 4:**
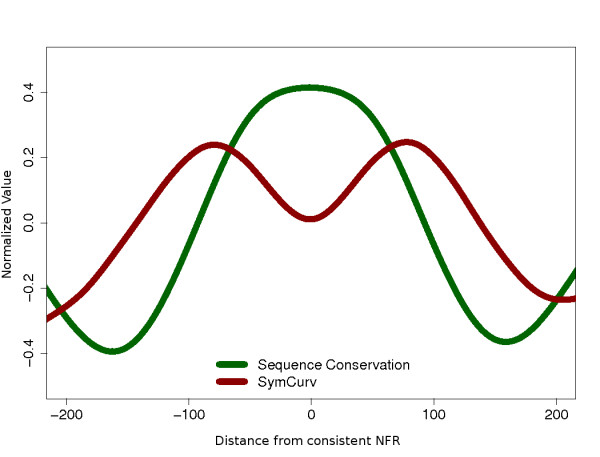
**Average conservation and SymCurv plotted around 1,099 consistent nucleosome-free regions (NFRs) defined through the comparison of two independent datasets (see text for details)**. The center of the NFR was set as zero. Both forward and reverse complements of each sequence were included in the analysis. Conservation was calculated as mean PhastCons scores over all sites with a specific distance from the centre of the NFR, and SymCurv calculated accordingly. Both curves were smoothed with a running average on a window of 50 bp for better representation.

Through the application of a recently introduced structural property of DNA [41] and a suitable measure of its variability developed in this study, we show that consistent nucleosomes have significantly different behavior compared with both weakly positioned (unstable) nucleosomes and NFRs, a difference that can be attributed only to specific constraints existing at the level of DNA structure. Such constraints may not necessarily be captured at the level of the primary sequence. In a recent work, Bettecken *et al*. [18] showed that the well-documented, nucleosome-related, dinucleotide periodicities drastically differ between organisms in terms of the dinucleotides involved. In their study of the *S. pombe *nucleosomal landscape, Lantermann *et al*. [44] reported significant discrepancies at the sequence level compared with the nucleosome sequence compositional preferences of *S. cerevisiae*. Such discrepancies at the DNA sequence level may disappear at structural level. In fact, dinucleotide structural properties such as roll, tilt and twist angles may shape the bendability, deformability and curvature of DNA molecules in a degenerate way. Different oligonucleotides may give rise to similar structures, which eventually are the elements to be recognized by DNA-binding proteins. Along the same lines, Travers *et al*. [45] support the view of a mixed deterministic-stochastic framework for nucleosome positioning, suggesting a 'tunable property' as the basis for the DNA-guided nucleosome positioning. Figure 4 gives a clear demonstration of how SymCurv may act as such a 'tunable property'. Normalized values of SymCurv and sequence conservation (as PhastCons) were plotted as an average over 1,099 centrally aligned consistent NFRs (see Results). The mirror-image-like pattern is suggestive of how sequences with low conservation may assume favorable conformations for the accommodation of nucleosomes. Some of the missing aspects of the complicated process of nucleosome positioning and the shaping of the underlying sequences may thus be revealed through the assessment of a simple structural property instead of with the use of a complicated model.

**Figure 5:**
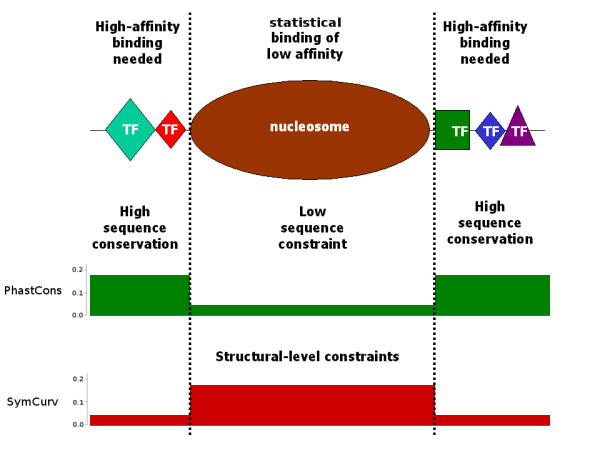
**A model for nucleosome positioning sequences**. Vertical dotted lines represent consistent nucleosome boundaries. Green and red filled plots with arbitrary axes are qualitative representations of sequence conservation and SymCurv, respectively, shown here to allow better description of the model (actual data presented in Figure 4). High-affinity binding is necessary for regulatory elements occupying the surrounding space and allowing nucleosomes to occupy regions of low sequence constraint. The consistent positioning of these nucleosomes does not require high affinity but imposes a secondary level of constraints of a structural type on the underlying sequences. The symmetry of DNA curvature may be seen as one constraint of this type.

In this work, we have attempted to use such a property to reconcile the existence of well-positioned 'key' nucleosomes with the lack of sequence conservation within them, by revealing a secondary type of constraint, emerging at the structural level. We show that the symmetry of DNA curvature, as quantified by SymCurv [41], is significantly higher in well-positioned nucleosomes compared with unstable, weakly positioned nucleosomal sequences. This second-level sequence pattern may be a reflection of a weaker type of constraint related to structural preferences of the histone octamer, which has been further fixed through the consistent positioning of nucleosomes in the same region through evolution. Our findings may thus be combined into a model for the emergence of a weak nucleosome-positioning code (Figure 5). According to this hypothesis, whose starting point is the statistical positioning of nucleosomes, consistent nucleosomes are only partly guided by nearby NFRs. Once established, a set of well-positioned nucleosomes may impose secondary constraints that further shape the underlying DNA. This 'molding' of the genomic sequence is not taking place at the level of the primary sequence but at that of secondary DNA structural conformation, as the only prerequisite is the accommodation of the histone octamer. Thus, in the same way that a sculptor creates a cast that bears no detailed characteristics of the sculpture other than its basic outlines, the DNA may be shaped in order for greater affinity with the octamer to be achieved, regardless of the primary sequence elements that constitute it.

Overall, our study suggests that sequence constraints related to nucleosome positioning, although weak and secondary, exist and are better reflected at the level of DNA structure. Defining the complete set of such constraints would therefore be of great value to our understanding of both chromatin organization and its role in gene regulation in eukaryotes.

## Methods

### Dataset comparison and *in vitro *validation

Genomic coordinate overlaps were calculated as described in the Results section. Validation was conducted using the *in vitro *data from Mavrich *et al*. [23]. For each nucleosome under examination, the corresponding segment of the Mavrich *et al*. [23] dataset that had an overlap > 0.95 was retained. Thus, three new subsets were formed, consisting of significant (≥ 0.95) overlaps of the Lee *et al*. [7] dataset, the Shivaswamy *et al*. [8] dataset and consistent nucleosomes, against the set of Mavrich *et al*. [23] (see Additional File 1).

Validation was performed with the *in vitro *data from Kaplan *et al*. [13]. For each set of genomic coordinates, we retrieved the model scores of Kaplan *et al*. [13]http://genie.weizmann.ac.il/software/data/Yeast_sacCer1_Model_Score.gxt.gz. As data were in the form of a single value per nucleotide, we calculated the sum of model scores and then averaged these over the length of the nucleosomal segment under examination (always equaling 147 nucleotides), resulting in one value representing the mean *in vitro *model score for each genomic region (see Additional File 1).

### Sequence conservation

To assess sequence conservation, we used an approach similar to that described above for the *in vitro *data. We obtained seven-way PhastCons values from UCSC Genome Browser http://hgdownload.cse.ucsc.edu/goldenPath/sacCer1/multizYeast/ for the complete yeast genome (2003 Assembly, sacCer1). PhastCons values were summed for each segment, and then averaged over its length. Averaged aggregates of these values for each genomic segment were used as a measure of its overall sequence conservation. Genomic coordinates were classified as genic or non-genic based on the genomic coordinates of SGD genes for the 2003 Assembly (sacCer1). Mean conservation (Figure 2, dotted lines) was calculated as the overall average of PhastCons values for all genic and all intergenic nucleotides.

#### Peaks

PEAKS [29] was implemented directly from the positional footprinting web server http://evolutionarygenomics.imim.es/PEAKS/usr/, using transcription factor binding sites matrices from a previous study [30], with a cut-off value of 0.99 and a footprinting window size of 11 nucleotides. Given a set of pre-aligned sequences, PEAKS detects motifs that show significant clustering at a particular distance from an arbitrary reference element, which in this case was set as the position of the pseudodyad axis of the assumed nucleosome.

To assess significance of positional biases, PEAKS uses random datasets to estimate a *P *value for each binding factor. In the graphical representation (Figure 3), each significant motif is represented as an oval, whose width corresponds to the range of the significant position, and whose height is the relative motif signal (RMS). The RMS is defined as the number of sequences that contain the motif in the region over the number of sequences that were expected to contain the motif at the *P*-value cut-off level (random expectance of the motif occurring in that region calculated for 1,000 random sets). Images produced by PEAKS are reproduced here with the permission of the program's authors.

### SymCurv: calculating the symmetry of curvature of a given DNA sequence

The symmetry of curvature of DNA sequences [41] was calculated by applying symmetry constraints on a DNA sequence's predicted curvature. Given a sequence on which curvature values are computed for each trinucleotide step, a symmetric pattern around a given nucleotide position *n *would imply similar values of curvature at equal distances from this position on either direction. That is, the value of curvature at position *n-1, Curv_n-1 _*should be similar to the value at position *n+1, Curv_n+1_*, and this should hold for all pairs of positions at distance *i *from *n *for *i = 1, ..., m*, where *m *is a suitably adjusted parameter. At each such distance, we can compute the absolute difference between the corresponding curvature values *d_i _= |Curv_n-i_-Curv_n+i_|*. The lower this value, the higher the symmetry within the given distance *i *from *n*.

We define the symmetry of the curvature of the sequence centered at position *n *on a window of length *m *as the inverse sum of the distances over all values from *1 *to *m:*

(1) $$S_{Sym}\left(m\right)=\sum_{i=\text{1}}^{m}\frac{\text{1}}{d_{i}}$$

The inversion in the *S_sym _*formula is performed to maximize the symmetry score; the more symmetric the values on either side of position *n*, the closer the sum of distances will approach zero and thus larger the symmetry value *S_sym _*will become. As values of *d_i _*are generally in the range of two orders of magnitude, we use the inversion in equation 1 (rather than, for example, the simpler use of its negative value) to increase the dynamic range of *S_sym _*values and thus 'spread' the *S_sym _*value range to better capture differences between sequences.

Based on the above definition of Symmetry, the symmetry of curvature is calculated as follows. Given a genomic sequence, the method proceeds by first calculating the curvature values and subsequently applying the symmetry constraints on the resulting curvature data.

First, curvature values of the given sequence are calculated. In the current version of our method, we calculate the DNA curvature using BENDS [46], extended with the use of trinucleotide parameters as described previously [47,48]. The output of this step is an array in which a curvature value is attributed to every nucleotide, calculated through a window of length of 30 bp centered on each nucleotide and sliding 1 bp at a time. According to this scheme, each trinucleotide and its reverse complement (for example, TAA/TTA) are equivalent in terms of structural parameters (roll, tilt and twist angles). It is thus an easy matter to apply the calculation to both the sequence under examination and its reverse complementary. This makes sense from the physical point of view as we expect that the nucleosome forming potential of a given DNA sequence be strand-independent.

Secondly, nucleosomal sequences have been reported [34,36,39,40] to be flexible around their central region, where local distortions are relaxed. Thus, the region of the pseudodyad axis may be expected to produce lower curvature values, separating two parts of overall higher curvature. A local curvature minimum is thus set as a prerequisite for a given site to be considered as a possible dyad axis, and the calculation of *S_sym _*is only to take place in sites fulfilling this condition. Thus, the curvature values array is scanned for local minima. For positions that fulfill the above criterion, a local minimum score is calculated according to the formula:

(2) $$S_{\min}(n)=\frac{1}{\left(Curv_{\left(n-1\right)}-Curv_{\left(n\right)})+(Curv_{\left(n+1\right)}-Curv_{\left(n\right)}\right)}$$

if *Curv*_(*n*-1) _>*Curv*_(*n*) _and *Curv*_(*n*-1) _>*Curv*_(*n*)_, while *S*_min _(*n*) = 0, otherwise *Curv_n _*is the curvature value at position *n *on the genomic sequence. The inversion in the *S_min _*formula is performed to selectively increase the scores for mild local minima, as the local decrease in curvature on the dyad axis region is expected to be a smooth, minor decrease rather than an acute one.

Thirdly, the *SymCurv *symmetry score at every local minimum site is calculated using equation 1. The length parameter *m *was set to 25, based on the combined size of the pseudodyad axis and the immediate flanking regions. The calculation is thus conducted over a window of 50 nucleotides, which corresponds to five DNA double-helical pitches. The overall score of the symmetry of curvature, SymCurv, is calculated as the product of the two scores.

(3) $$SymCurv_{\left(n,m\right)}=S_{min(n)}S_{sym(n,m)},$$

where *m *= 25.

We should note here that use of *m *values in the range of (*m *= 15 to 35), corresponding to three to seven helical turns yielded similar results. We therefore chose to use *m *= 25 (~five helical turns) as it is closer to the known size of the dyad axis region [49] and its immediate flanking sequences, which have been shown to be contributing the most to histone binding [50,51].

SymCurv thus assigns a value for each nucleotide. Given a region of size *L *nucleotides, we may calculate an overall SymCurv value for the genomic segment as the average over all nucleotides.

(4) $$SymCurv\left(L\right)=\sum_{i}^{L}\frac{SymCurv\left(i\right)}{L}$$

We proceeded in such a way to compute SymCurv values for nucleosome and NFR regions in this study. To allow direct comparison, the SymCurv values in Figures 3b and Figure 4 were normalized in the form of z-scores:

(5) $$ZSC[i]=\frac{SC[i]-mean(SC)}{sd(SC)},$$

where the new ZSC[i] value was calculated on the basis of mean and standard deviation values of SymCurv for the complete yeast genome. Thus, the average values were set around 0.

#### Robustness of SymCurv

The robustness of SymCurv values was then tested. Given a DNA sequence of length *L*, its average SymCurv value was initially calculated as described above. We then produced the complete set of DNA sequences, which differ from the original by one nucleotide, by mutating all individual positions but keeping the rest of the sequence intact. For a sequence of length *L*, there are 3*L *one-nucleotide mutants or neighbors. We then calculated the average SymCurv values for all neighbors and defined the distance within them as the variance of the four values for each nucleotide position. Thus for nucleotide *i *the distance *D*(*i*) is:

D(i)=var(SymCurv[A],SymCurv[G],SymCurv[C],SymCurv[T]),

where *SymCurv[X] *is the value of SymCurv at the *i*-th nucleotide for the neighbor bearing nucleotide *X *at that specific position.

The overall distance for the complete sequence *D_seq _*is then calculated as the average over all *L *positions:

$$D_{seq}=\sum_{i}^{L}\frac{D\left(i\right)}{L}$$

As high variance is a measure of variability, which is inversely related to robustness, we may define robustness (*R*) as the negative logarithm of the above distance:

$$R_{seq}=-\text{log}\left(D_{seq}\right)$$

The logarithm is used here to decrease the dynamic range of *R *purely for practical reasons, as overall distances (*D*) exhibit a range of values over several orders of magnitude.

A property such as *R_seq _*is used as a measure of the variance of the SymCurv values between the one-nucleotide neighbors. It represents the tendency of a given sequence to radically alter its structural properties (as measured by SymCurv) given a single mutation anywhere within it. In this sense, robust sequences will tend to have low (strongly negative) values of *D*. By contrast, sequences under strong structural constraints will tend to have increased variance, as even single nucleotide mutations may bring about notable changes in the structural profile, and their robustness will therefore be decreased.

## Competing interests

The authors declare that they have no competing interests.

## Authors' contributions

CN conceived the study, performed the SymCurv analysis, suggested the model and wrote the paper. SA carried out the computational analyses. MB coordinated the project and reviewed the manuscript. RG suggested the study, coordinated the project, designed the analyses, and reviewed the manuscript. All authors read and approved the final manuscript.

## Supplementary Material

Additional file 1 **Additional information on the definition and validation of consistent nucleosomes**. Text containing additional information on the definition and validation of consistent nucleosomes. Click here for file

## References

[B1] Guenther MG Levine SS Boyer LA Jaenisch R Young RA A chromatin landmark and transcription initiation at most promoters in human cells Cell 2007 130 77 88 10.1016/j.cell.2007.05.042 17632057 PMC3200295

[B2] Mellor J Dynamic nucleosomes and gene transcription Trends in Genetics 2006 22 320 329 10.1016/j.tig.2006.03.008 16631276

[B3] Eaton ML Galani K Kang S Bell SP MacAlpine DM Conserved nucleosome positioning defines replication origins Genes & development 2010 24 748 10.1101/gad.1913210 PMC2854390 20351051

[B4] Yin S Deng W Hu L Kong X The impact of nucleosome positioning on the organization of replication origins in eukaryotes Biochemical and Biophysical Research Communications 2009 385 363 368 10.1016/j.bbrc.2009.05.072 19463783

[B5] Vicent GP Nacht AS Smith CL Peterson CL Dimitrov S Beato M DNA instructed displacement of histones H2A and H2B at an inducible promoter MOLECULAR CELL 2004 16 439 452 10.1016/j.molcel.2004.10.025 15525516

[B6] Yuan GC Liu YJ Dion MF Slack MD Wu LF Altschuler SJ Rando OJ Genome-scale identification of nucleosome positions in S. cerevisiae Science 2005 309 626 10.1126/science.1112178 15961632

[B7] Lee W Tillo D Bray N Morse RH Davis RW Hughes TR Nislow C A high-resolution atlas of nucleosome occupancy in yeast Nature Genetics 2007 39 1235 1244 10.1038/ng2117 17873876

[B8] Shivaswamy S Bhinge A Zhao Y Jones S Hirst M Iyer VR Dynamic remodeling of individual nucleosomes across a eukaryotic genome in response to transcriptional perturbation PLoS Biol 2008 6 65 10.1371/journal.pbio.0060065 PMC2267817 18351804

[B9] Stein A Takasuka TE Collings CK Are nucleosome positions in vivo primarily determined by histone-DNA sequence preferences? Nucleic Acids Research 2009 10.1093/nar/gkp1043 PMC2817465 19934265

[B10] Feng J Dai X Xiang Q Dai Z Wang J Deng Y He C New insights into two distinct nucleosome distributions: comparison of cross-platform positioning datasets in the yeast genome BMC Genomics 2010 11 10.1186/1471-2164-11-33 PMC2824721 20078849

[B11] Caserta M A translational signature for nucleosome positioning in vivo Nucleic Acids Research 2009 19596807 10.1093/nar/gkp574 PMC2760819

[B12] Ioshikhes IP Albert I Zanton SJ Pugh BF Nucleosome positions predicted through comparative genomics Nature 2006 200 6 10.1038/ng1878 16964265

[B13] Kaplan N Moore IK Fondufe-Mittendorf Y Gossett AJ Tillo D Field Y LeProust EM Hughes TR Lieb JD Widom J The DNA-encoded nucleosome organization of a eukaryotic genome Nature 2008 458 362 366 10.1038/nature07667 19092803 PMC2658732

[B14] Ogawa R Kitagawa N Ashida H Saito R Tomita M Computational prediction of nucleosome positioning by calculating the relative fragment frequency index of nucleosomal sequences FEBS Letters 2010 584 1498 1502 10.1016/j.febslet.2010.02.067 20206172

[B15] Peckham HE Thurman RE Fu Y Stamatoyannopoulos JA Noble WS Struhl K Weng Z Nucleosome positioning signals in genomic DNA Genome Research 2007 17 1170 10.1101/gr.6101007 17620451 PMC1933512

[B16] Segal E Fondufe-Mittendorf Y Chen L Thastrom AC Field Y Moore IK Wang JPZ Widom J A genomic code for nucleosome positioning Nature 2006 442 772 778 10.1038/nature04979 16862119 PMC2623244

[B17] Babbitt G Tolstorukov M Kim Y The molecular evolution of nucleosome positioning through sequence-dependent deformation of the DNA polymer Journal of Biomolecular Structure & Dynamics 2010 27 765 10.1080/07391102.2010.10508584 20232932

[B18] Bettecken T Trifonov EN Seoighe C Repertoires of the Nucleosome-Positioning Dinucleotides PloS one 2009 4 e7654 10.1371/journal.pone.0007654 19888331 PMC2765632

[B19] Chung HR Vingron M Sequence-dependent nucleosome positioning Journal of molecular biology 2009 386 1411 1422 10.1016/j.jmb.2008.11.049 19070622

[B20] Fraser RM Keszenman-Pereyra D Simmen MW Allan J High-resolution mapping of sequence-directed nucleosome positioning on genomic DNA Journal of Molecular Biology 2009 390 292 305 10.1016/j.jmb.2009.04.079 19427325

[B21] Kornberg R The location of nucleosomes in chromatin: specific or statistical? Nature 1981 292 579 580 10.1038/292579a0 7254354

[B22] Kornberg RD Stryer L Statistical distributions of nucleosomes: nonrandom locations by a stochastic mechanism Nucleic Acids Research 1988 16 6677 6690 10.1093/nar/16.14.6677 3399412 PMC338322

[B23] Mavrich TN Ioshikhes IP Venters BJ Jiang C Tomsho LP Qi J Schuster SC Albert I Pugh BF A barrier nucleosome model for statistical positioning of nucleosomes throughout the yeast genome Genome research 2008 18 1073 10.1101/gr.078261.108 18550805 PMC2493396

[B24] Zhang Y Moqtaderi Z Rattner BP Euskirchen G Snyder M Kadonaga JT Liu XS Struhl K Intrinsic histone-DNA interactions are not the major determinant of nucleosome positions in vivo Nature structural & molecular biology 2009 10.1038/nsmb.1636 PMC2823114 19620965

[B25] Reynolds SM Bilmes JA Noble WS Learning a Weighted Sequence Model of the Nucleosome Core and Linker Yields More Accurate Predictions in *Saccharomyces cerevisiae *and *Homo sapiens* PLoS Computational Biology 2008 6 e1000834 10.1371/journal.pcbi.1000834 PMC2900294 20628623

[B26] Siepel A Bejerano G Pedersen JS Hinrichs AS Hou M Rosenbloom K Clawson H Spieth J Hillier LDW Richards S others Evolutionarily conserved elements in vertebrate, insect, worm, and yeast genomes Genome research 2005 15 1034 10.1101/gr.3715005 16024819 PMC1182216

[B27] Milani P Chevereau G Vaillant C Audit B Haftek-Terreau Z Marilley M Bouvet P Argoul F Arneodo A Nucleosome positioning by genomic excluding-energy barriers Proceedings of the National Academy of Sciences 2009 106 22257 10.1073/pnas.0909511106 PMC2799728 20018700

[B28] Henikoff S Labile H3. 3+ H2A. Z nucleosomes mark 'nucleosome-free regions' Nature genetics 2009 41 865 866 10.1038/ng0809-865 19639024

[B29] Bellora N Farre D Mar Alba M PEAKS: identification of regulatory motifs by their position in DNA sequences Bioinformatics 2007 23 243 10.1093/bioinformatics/btl568 17098773

[B30] Harbison CT Gordon DB Lee TI Rinaldi NJ Macisaac KD Danford TW Hannett NM Tagne JB Reynolds DB Yoo J others Transcriptional regulatory code of a eukaryotic genome Nature 2004 431 99 104 10.1038/nature02800 15343339 PMC3006441

[B31] Cohanim AB Haran TE The coexistence of the nucleosome positioning code with the genetic code on eukaryotic genomes Nucleic Acids Research 2009 37 6466 10.1093/nar/gkp689 19700771 PMC2770662

[B32] Johnson SM Tan FJ McCullough HL Riordan DP Fire AZ Flexibility and constraint in the nucleosome core landscape of *Caenorhabditis elegans *chromatin Genome research 2006 16 1505 10.1101/gr.5560806 17038564 PMC1665634

[B33] Valouev A Ichikawa J Tonthat T Stuart J Ranade S Peckham H Zeng K Malek JA Costa G McKernan K others A high-resolution, nucleosome position map of *C. elegans *reveals a lack of universal sequence-dictated positioning Genome research 2008 18 1051 10.1101/gr.076463.108 18477713 PMC2493394

[B34] Belikov S Kapranov A Karpov V Uncurved-DNA signals are important for translational positioning of nucleosomes J Biomol Struct Dyn 1997 15 625 630 9440009 10.1080/07391102.1997.10508973

[B35] Drew HR Travers AA DNA bending and its relation to nucleosome positioning Journal of molecular biology 1985 186 773 790 10.1016/0022-2836(85)90396-1 3912515

[B36] Edayathumangalam RS Weyermann P Gottesfeld JM Dervan PB Luger K Molecular recognition of the nucleosomal supergroove Proceedings of the National Academy of Sciences of the United States of America 2004 101 6864 10.1073/pnas.0401743101 15100411 PMC406433

[B37] Richmond TJ Davey CA The structure of DNA in the nucleosome core Nature 2003 423 145 150 10.1038/nature01595 12736678

[B38] Battistini F Hunter CA Gardiner EJ Packer MJ Structural Mechanics of DNA Wrapping in the Nucleosome Journal of molecular biology 2010 396 264 279 10.1016/j.jmb.2009.11.040 19932116

[B39] Hayes JJ Clark DJ Wolffe AP Histone contributions to the structure of DNA in the nucleosome Proceedings of the National Academy of Sciences 1991 88 6829 10.1073/pnas.88.15.6829 PMC52182 1650485

[B40] Ramsay N Deletion analysis of a DNA sequence that positions itself precisely on the nucleosome core Journal of molecular biology 1986 189 179 188 10.1016/0022-2836(86)90389-X 3783673

[B41] Tilgner H Nikolaou C Althammer S Sammeth M Beato M Valcarcel J Guigo R Nucleosome positioning as a determinant of exon recognition Nature structural & molecular biology 2009 16 996 1001 10.1038/nsmb.1658 19684599

[B42] Mobius W Gerland U Segal E Quantitative test of the barrier nucleosome model for statistical positioning of nucleosomes up-and downstream of transcription start sites PLoS Computational Biology 2010 6 10.1371/journal.pcbi.1000891 20808881 PMC2924246

[B43] Kiyama R Trifonov EN What positions nucleosomes?-A model FEBS letters 2002 523 7 11 10.1016/S0014-5793(02)02937-X 12123795

[B44] Lantermann AB Straub T Stralfors A Yuan GC Ekwall K Korber P *Schizosaccharomyces pombe *genome-wide nucleosome mapping reveals positioning mechanisms distinct from those of *Saccharomyces cerevisiae* Nature structural & molecular biology 2010 17 251 257 10.1038/nsmb.1741 20118936

[B45] Travers A Hiriart E Churcher M Caserta M Di Mauro E The DNA Sequence-dependence of Nucleosome Positioning in vivo and in vitro Journal of biomolecular structure & dynamics 2010 27 713 10.1080/073911010010524942 PMC2864905 20232928

[B46] Goodsell DS Dickerson RE Bending and curvature calculations in B-DNA Nucleic acids research 1994 22 5497 10.1093/nar/22.24.5497 7816643 PMC332108

[B47] Brukner I S nchez R Suck D Pongor S Sequence-dependent bending propensity of DNA as revealed by DNase I: parameters for trinucleotides The EMBO journal 1995 14 1812 7737131 10.1002/j.1460-2075.1995.tb07169.x PMC398274

[B48] Munteanu MG Vlahovicek K Parthasarathy S Simon I Pongor S Rod models of DNA: sequence-dependent anisotropic elastic modelling of local bending phenomena Trends in Biochemical Sciences 1998 23 341 347 10.1016/S0968-0004(98)01265-1 9787640

[B49] Arents G Burlingame RW Wang BC Love WE Moudrianakis EN The nucleosomal core histone octamer at 3.1 A resolution: a tripartite protein assembly and a left-handed superhelix Proceedings of the National Academy of Sciences of the United States of America 1991 88 10148 10.1073/pnas.88.22.10148 1946434 PMC52885

[B50] Thastrom A Lowary PT Widlund HR Cao H Kubista M Widom J Sequence motifs and free energies of selected natural and non-natural nucleosome positioning DNA sequences1 Journal of molecular biology 1999 288 213 229 10.1006/jmbi.1999.2686 10329138

[B51] Widlund HR Cao H Simonsson S Magnusson E Simonsson T Nielsen PE Kahn JD Crothers DM Kubista M Identification and characterization of genomic nucleosome-positioning sequences Journal of molecular biology 1997 267 807 817 10.1006/jmbi.1997.0916 9135113

