# Supplementary material for: Structural constraints revealed in consistent nucleosome positions in the genome of S. cerevisiae
Source: Epigenetics Chromatin. 2010 Nov 12;3:20. doi: 10.1186/1756-8935-3-20 (PMC2994855; doi:10.1186/1756-8935-3-20)
Supplement: Additional file 1 — Additional information on the definition and validation of consistent nucleosomes. Text containing additional information on the definition and validation of consistent nucleosomes. [file 1756-8935-3-20-S1.DOC]

Additional File 1

Structural constraints revealed in consistent nucleosome positions in the genome of *S. cerevisiae*

Christoforos Nikolaou^1^, Sonja Althammer^1^, Miguel Beato^2^ and Roderic Guigó^1^

*^1^Bioinformatics and Genomics Group,*

*^2^ Gene Regulation and Chromatin Group,*

*The Centre for Genomic Regulation (CRG),*

*Biomedical Research Park of Barcelona (PRBB), Barcelona, 08003, Catalunya, Spain*

***Consistent nucleosomes***

We defined consistent nucleosomes in the following way.

We connected each of the experimental calls of the dataset of Lee et al. [1] (40089 nucleosomes) with its closest corresponding nucleosome of the dataset of of Shivaswamy et al. [2] (49043 nucleosomes). For every such pair we calculated the percentage of overlap, expressed as the ratio of the intersection over the union of their corresponding lengths. We then analyzed the distribution of these overlaps (Supplementary Figure 1). We found that 31234 nucleosomes of the Lee et al. [1] dataset had at least 1 nucleotide overlap with the set of Shivaswamy et al [2] . Among these overlapping positions only 9.8% of the cases (3061 nucleosomes) had an overlap, which exceeded 0.95 while 17.8% of the cases (5560 nucleosomes) had an overlap greater than 0.90. To test the significance of these values we performed 1000 simulations of two random experiments which produced equal number of nucleosomal calls. The overlap values between two random experiments followed a Poisson distribution, which was significantly different from the observed one (p-value<10^-6^ according to a Kolmogorov-Smirnov test). In fact, an overlap ratio of 0.95 was not encountered in any of the 1000 simulations. We thus opted for this rather strict threshold of 0.95 to define a subset of consistent nucleosome positions among the two datasets.

**
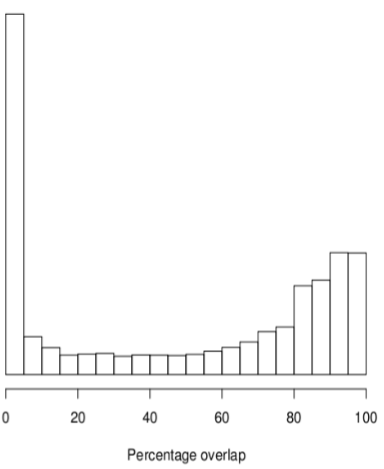
**

***Supplementary Figure 1. Percentage of overlap between datasets of Lee et al. [1] and of Shivaswamy et al [2] .***

*Bimodal distribution of overlap ratios (bar histogram) shows clear discrepancy with the expected distribution under assumption of two random distributions of equal number of segments (blue line).*

***Consistent nucleosomes are well positioned in vivo and in vitro***

Initial validation of the consistent nucleosomes was conducted through direct comparison with *in vivo* and *in vitro* hybridization scores. In particular, a direct comparison of the two previously discussed datasets as well as their overlapping subset was then cross-validated using the scores of experimental accuracy as provided by Shivaswamy et al. [2], Mavrich et al. [3] and Kaplan et al. [4]. In the first case the scores represented a measure of the consistency of occupancy of the given segment by a nucleosome, roughly speaking how often this region was found to be occupied by a nucleosome in the experimental replicates. We used the initial *in vivo* scores provided by Shivaswamy et al. [2] for each of their nucleosome calls. Scores for nucleosomes with no overlap between [1] and [2] showed significantly lower scores than those with high overlap values (Supplementary Figure 2). Since these scores were directly linked to the initial raw experimental signal, the observed differences may come as an indication that consistent nucleosomes are positioned more strongly *in vivo*.


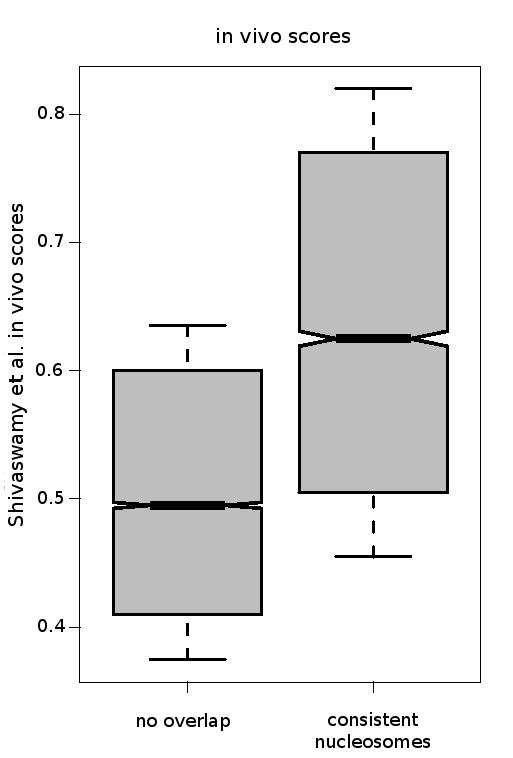


***Supplementary Figure 2.*** *In vivo scores as provided by Shivaswamy et al. [2] for no overlapping nucleosomes against consiststent nucleosomes. (distributions different with a p-value<10^-16^)*

Mavrich et al. [3] also provide a score for each inferred nucleosome position in their dataset. This is an occupancy measure based on the model-normalized occupancy across all of their four sequencing datasets, ranging from 0 to 100. In this sense a mean-score of 100 represents positions found in all four replicate datasets. To further validate our dataset of consistent nucleosomes we obtained the cases that had more than 0.95 overlap with the dataset of Mavrich et al. [3] (~66000 nucleosomes) and plotted the distribution of mean occupancy based on their calculations (Supplementary Figure 3). We saw that mean occupancy gradually increased as we moved from the complete dataset of Mavrich et al. [3] to two-set overlaps, reaching a mean score of 93 in the case of the three-set overlap (segments of Lee et al., Shivaswamy et al. and Mavrich et al. overlapping in more than 0.95 of their lengths). In fact, none of the 3061 consistent nuclesomes, defined as the overlaps between [1] and [2] showed an overlap lesser than 0.96, which came as an additional indication of this dataset comprising a subset of highly reproducible positions.


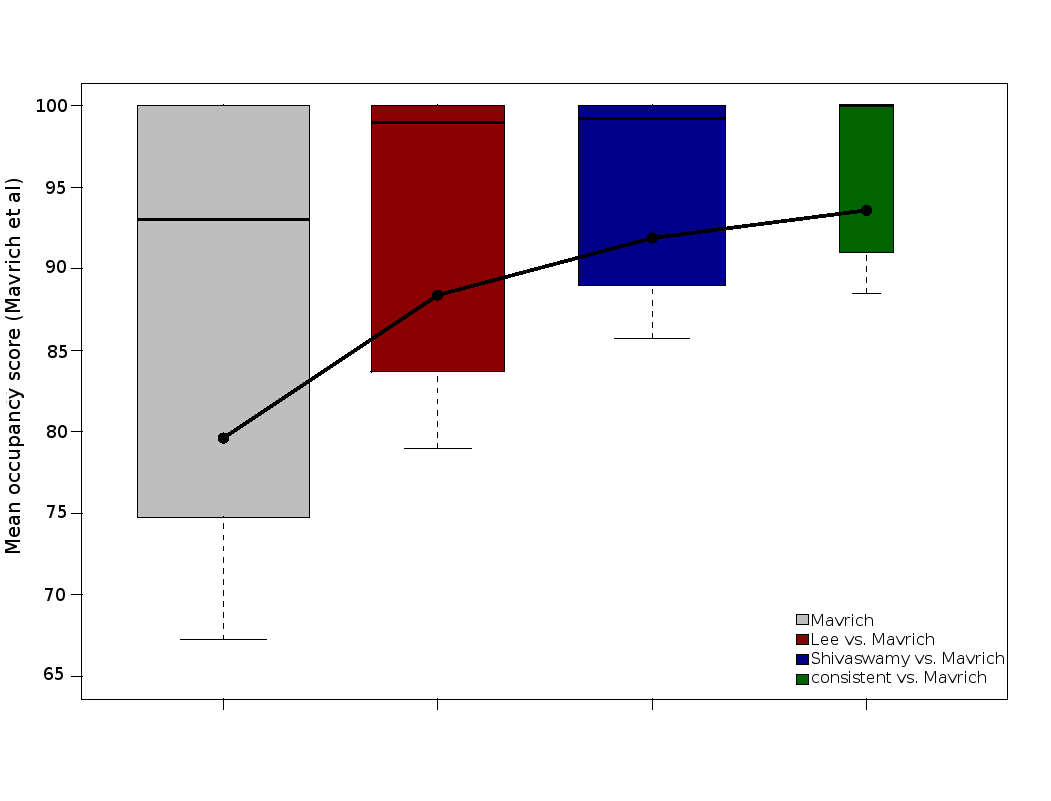


***Supplementary Figure 3.*** *Mean nucleosome occupancy as calculated by Mavrich et al. [3] based on the consistency of nucleosome positions among 4 experimental replicates. Nucleosomes that coincide between different datasets show increasing mean occupancy. Nucleosomes coinciding in all three datasets represent the most consistent subset. Black line represents mean occupancies for the 4 datasets*

In a recent work, Kaplan et al. [4] have attempted to decouple the intrinsic sequence preferences of nucleosomes from the combined action of all influencing factors and have thus provided a measure for the *in vitro* affinity of the underlying DNA for nucleosome formation. Although this is expected to correlate well with in vivo positioning, we went on to examine possible differences between consistent and bulk nucleosomes. We found an expected significant enrichment of Kaplan et al. [4] model scores for consistent nucleosomes when compared to both bulk (total) nucleosomes and non-overlapping ones (Supplementary Figure 4).


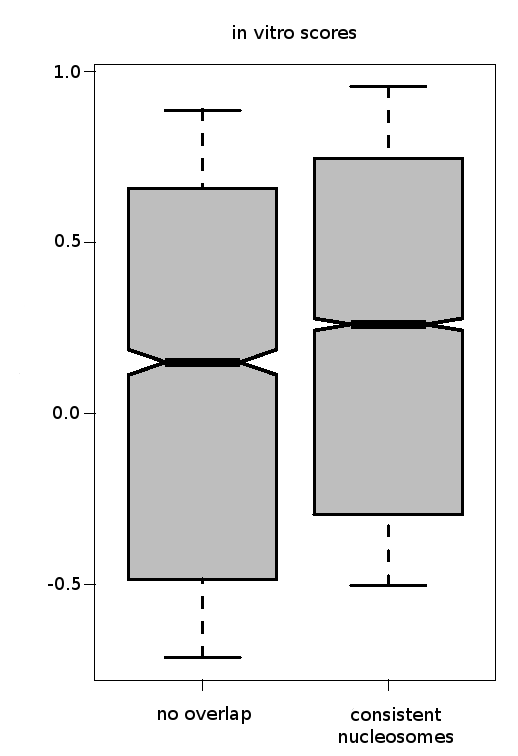


***Supplementary Figure 4.*** *In vitro scores as provided by Kaplan et al. [4] for no overlapping nucleosomes against consiststent nucleosomes. Score were calculated as mean-aggregates of raw scores provided at nucleotide resolution (distributions different with a p-value<10^-11^) (see text for details)*

***Positional preferences of consistent nucleosomes***

We analyzed the positional distribution of “consistent” nucleosomes along the genome of S. cerevisiae. This distribution is non-random and particularly biased towards promoter regions and sequences flanking the transcription start sites of genes. Both, Lee et al.[1], and Shivaswamy et al. [2] , as well as previous [5] and more recent [6] papers have pointed out the enrichment of nucleosomes in genes as opposed to intergenic regions. We calculated the average enrichment of nucleosomes in non-genic versus genic regions for the two studies [1, 2] separately as well as for the subset of consistent nucleosomes. The results show an average enrichment of the consistent nucleosomes in non-genic regions that is more than 2-fold when compared to the bulk sets of nucleosomes in each individual experiment (Supplementary Figure 5a). Since genes represent the majority of the yeast genome, this suggests a clear preference for the consistent nucleosomes to occupy intergenic regions. We analyzed this feature in more detail by restricting the analysis in gradually narrowing regions around the transcription initiation sites of genes. While no enrichement of bulk nucleosomes is detected as we approach the TSS, a clear enrichment is detected of consistent nucleosomes (Supplementary Figure 5b). The above findings suggest that, in contrast to the overall greater density of bulk nucleosomes, well-positioned nucleosomes have a preference for intergenic regions—and specifically, for gene upstream regions proximal to the transcription start sites of genes. This finding is in agreement with a model in which nucleosomes positioned at the beginning of genes functioning as barriers [3, 7]. A consistently positioned nucleosome close to the TSS is more likely to be functioning as an organizing agent in the subsequent statistical positioning of the remaining nucleosomes, as this model assumes.

Interestingly, consistent nucleosomes appear to cluster in the genome, being di-nucleosomes particularly prominent. Indeed, Supplementary Figure 5c shows the distribution of distances between consistent nucleosomes. A clear peak appears at around 180bp, which considering an average inter-nucleosomal linker of 25-30nts [1, 2, 5] , would correspond to di-nucleosome clusters. In fact, almost 20% of consistent nucleosomes have a center-to-center distance shorter than 300nts in length (compared to 2% expected by chance, p-value < 10-6). The enrichment of linker lengths, which fall shorter than the size of a nucleosome, strongly suggests over-representation of di-nucleosomes among consistent nucleosomes. A possible explanation for this clustering could be the organizational properties of the linker sequence. As we discuss in the main paper, and as others [5, 6, 8] have also noticed under certain circumstances the regions between nucleosomes may be playing an important role in their positioning. A second main peak around ~3000bp falls very close to the average gene-to-gene distance in yeast, thus corresponding to consistent nucleosomes positioned with a periodical preference at the proximity of transcription initiation sites.

**
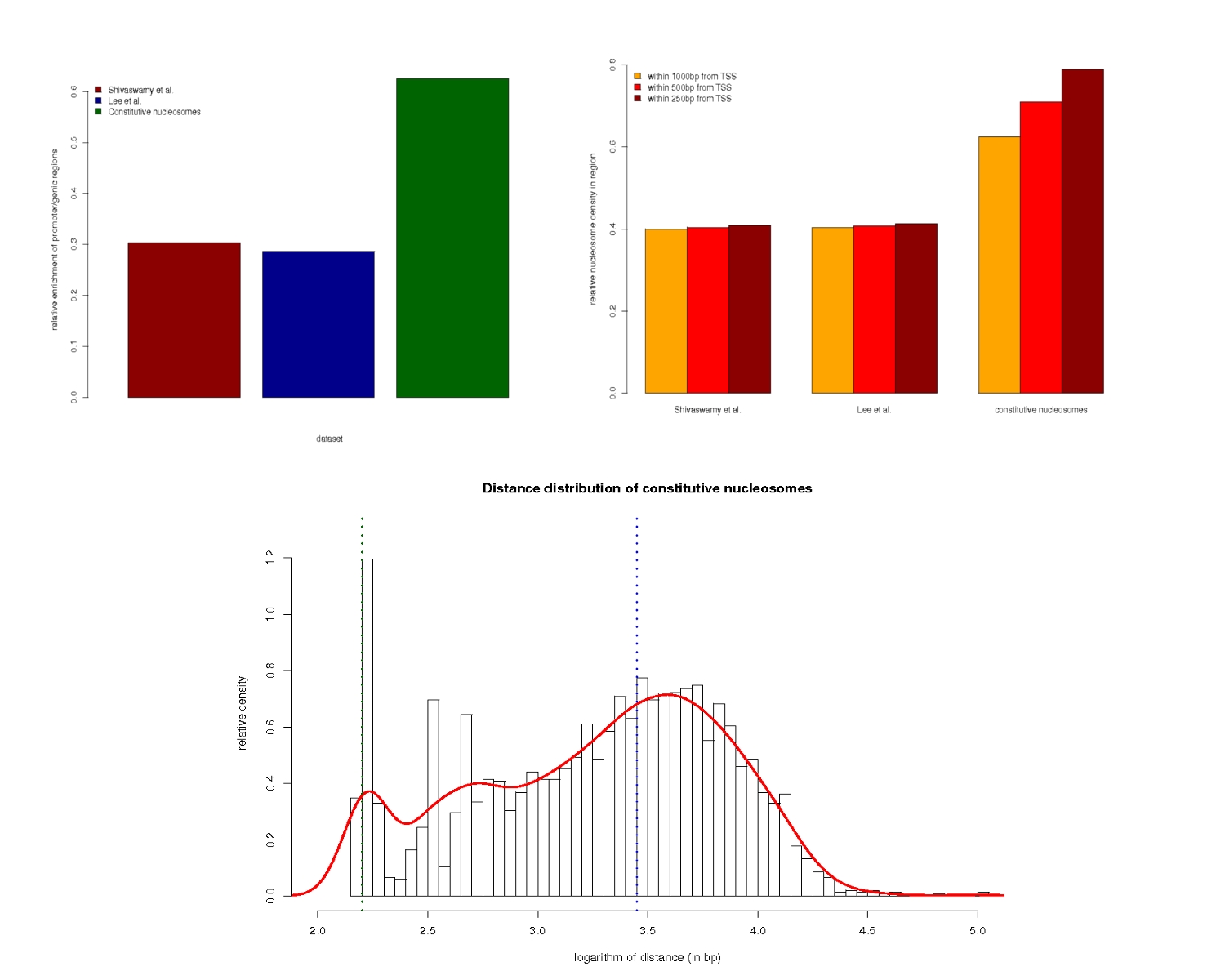
**

**Supplementary Figure 5. Positional Preferences of Consistent Nucleosomes**

*Intergenic regions are enriched in consistent nucleosomes. The relative percentage of nucleosomes in gene-upstream/genic regions is increased more than 2-fold when one considers the subset of nucleosomes that occupy the same positions in the two independent genome-scale experiments. Relative enrichment calculated as the ratio of nucleosomes found in promoters (limited up to 500nts upstream) against those found within genes, normalized over the total size of each partition b) Consistent nucleosomes tend to be positioned at the boundaries of genic-intergenic regions. As the margin around the transcription start site is narrowed the relative density of nucleosomes is gradually increased. Relative nucleosome density calculated as the percentage of nucleosomes located in the region divided by the length of the region. c) Distribution of centre-to-centre distances of consistent nucleosomes. Two main peaks correspond to a) the average size of an inter-nucleosomal linker (green dotted line) and b) the average size of a gene locus (blue dotted line) suggesting the over-representation of di-nucleosomes and the regular spacing of consistent nucleosome at gene boundaries.*

**References**

1. Lee W, Tillo D, Bray N, Morse RH, Davis RW, Hughes TR, Nislow C: **A high-resolution atlas of nucleosome occupancy in yeast.** *Nature genetics* 2007, **39:**1235-1244.

2. Shivaswamy S, Bhinge A, Zhao Y, Jones S, Hirst M, Iyer VR: **Dynamic remodeling of individual nucleosomes across a eukaryotic genome in response to transcriptional perturbation.** *PLoS Biol* 2008, **6:**65.

3. Mavrich TN, Ioshikhes IP, Venters BJ, Jiang C, Tomsho LP, Qi J, Schuster SC, Albert I, Pugh BF: **A barrier nucleosome model for statistical positioning of nucleosomes throughout the yeast genome.** *Genome research* 2008, **18:**1073.

4. Kaplan N, Moore IK, Fondufe-Mittendorf Y, Gossett AJ, Tillo D, Field Y, LeProust EM, Hughes TR, Lieb JD, Widom J: **The DNA-encoded nucleosome organization of a eukaryotic genome.** *Nature* 2008, **458:**362-366.

5. Yuan GC, Liu YJ, Dion MF, Slack MD, Wu LF, Altschuler SJ, Rando OJ: **Genome-scale identification of nucleosome positions in S. cerevisiae.** *Science* 2005, **309:**626.

6. Reynolds SM, Bilmes JA, Noble WS: **Learning a Weighted Sequence Model of the Nucleosome Core and Linker Yields More Accurate Predictions in <italic>Saccharomyces cerevisiae</italic> and <italic>Homo sapiens</italic>.** *PLoS Computational Biology* 2008, **6:**e1000834.

7. Zhang Y, Moqtaderi Z, Rattner BP, Euskirchen G, Snyder M, Kadonaga JT, Liu XS, Struhl K: **Intrinsic histone-DNA interactions are not the major determinant of nucleosome positions in vivo.** *Nature structural & molecular biology* 2009.

8. Segal E, Fondufe-Mittendorf Y, Chen L, Thastrom AC, Field Y, Moore IK, Wang JPZ, Widom J: **A genomic code for nucleosome positioning.** *Nature* 2006, **442:**772-778.
